# Maturity and density of tertiary lymphoid structures associate with tumor metastasis and chemotherapy response

**DOI:** 10.3389/fmed.2024.1435620

**Published:** 2024-10-18

**Authors:** Sutian Jiang, Xuhui Liao, Xuzhong Ding

**Affiliations:** ^1^Department of Pathology, Lishui People’s Hospital, Lishui, China; ^2^Department of Gastrointestinal Surgery, Lishui People’s Hospital, Lishui, China

**Keywords:** TLSs, outcomes, distant metastasis, adjuvant chemotherapy, gastric cancer

## Abstract

**Background:**

Tertiary Lymphoid Structures (TLSs) are abnormal clusters of immune cells that form in tissues not normally associated with the immune system, usually in cases of long-lasting inflammation, like cancer. TLSs have been suggested as a potential prognostic indicator in various cancer types.

**Methods:**

We retrospectively enrolled 223 gastric cancer (GC) patients who had surgical resections in this study. We utilized hematoxylin and eosin (HE) staining to detect the presence, abundance, and maturity of TLSs. In serial sections, we used immunohistochemistry to examine the cellular composition of TLSs.

**Results:**

The pathological review identified TLSs in 95.1% of the tumors, lymphoid aggregates in 79.8%, primary follicles in 45.7%, and lymphoid aggregates in 95.1% of the cases. Based on Kaplan-Meier curves, the maturation and abundance of TLSs contributed to longer disease-free survival (DFS) and overall survival (OS). In addition, the density of TLSs was strongly associated with the occurrence of tumor metastases and the response to adjuvant chemotherapy.

**Conclusions:**

We validated the prognostic value of TLSs in GC patients in both independent cohorts, and the maturity and density of TLS correlated with tumor metastasis. In addition, TLS may reflect sustained antitumor potency, which has important implications for adjuvant chemotherapy.

## Introduction

Gastric cancer (GC) is a common cancer worldwide, and despite improvements in treatment approaches, its prognosis remains unfavorable (1). The diversity observed in gastric cancer, both on a molecular and clinical level, presents significant obstacles in accurately predicting patient outcomes and identifying potential targets for therapy (2, 3). Despite its complex functions in various disease conditions, the immune microenvironment is widely acknowledged as a crucial controller in all types of cancer (4). Postnatal, organized clusters of immune cells known as Tertiary Lymphoid Structures (TLSs) develop in non-lymphoid tissues (5). TLS is made up of a T cell zone with mature dendritic cells (DCs), a germinal center with proliferating B cells, follicular dendritic cells, and high endothelial venules (6). Chronic inflammatory conditions, such as autoimmune diseases, chronic infections, and cancer, are where TLS was found, not physiological conditions (7, 8). In most cases, the occurrence of TLSs in tumors was associated with a positive prognosis and effective clinical outcomes of immunotherapy. In the tumor environment, TLS promoted immune cell infiltration into solid tumors, and thus the development of TLS was significantly associated with survival in untreated patients (9). Similarly, in patients treated with immune checkpoint inhibitors, the development of TLS was usually associated with an improved response to therapy (10). This suggested the conjecture that TLS was generating anti-tumor immune loci. Consequently, its use as a means of enhancing patients’ anti-tumor immunity and favoring therapeutic response has attracted widespread interest (5). Nevertheless, despite the apparent significance of TLSs, the factors behind their development in cancer and the role of these formations in the immune response within the tumor were still not well comprehended (7, 11).

In our findings, we retrospectively collected clinicopathologic information, prognostic information, and therapeutic strategies from a group of GC patients. Additionally, we conducted an investigation using immunohistochemistry (IHC) to assess the presence and characteristics of TLS in GC tissues. Specialized pathologists systematically assessed the distribution of the number and type of TLSs in each patient’s sample and performed a composite score. We hypothesized that the spatial organization of TLS may serve as a predictive marker of prognosis and was essential to improve patient responsiveness to adjuvant chemotherapy.

## Materials and methods

### Samples collection

We retrospectively collected 223 paraffin-embedded gastric cancer (GC) tissue sections at the Department of Pathology, Lishui People’s Hospital between 2016 and 2018. Clinicopathological characteristics, disease-free survival, and overall survival were also collected retrospectively. We collected clinicopathologic characteristics including age, sex, degree of differentiation, pathologic T status, pathologic N status, pathologic M status, vascular invasion, lymphatic invasion, surgical approach, distant metastatic site, adjuvant chemotherapy status, and P53 positivity. The study also included H&E stained data and clinical information of 408 GC patients from the Cancer Genome Atlas (TCGA) dataset^1^ (12). This research protocol was approved by the Human Research Ethics Committee of Lishui People’s Hospital (Zhejiang, China).

### H&E staining

The paraffin-embedded GC tissue specimens were sliced into 4 μm consecutive sections (13). These sections underwent dewaxing and rehydration using xylene and ethanol, respectively. Subsequently, the specimens were stained using hematoxylin for 30 s and then exposed to 1% acid ethanol for 3 s. The sections were washed in purified water and treated with eosin for a duration of 3 min. Following that, dehydration and hyalinization procedures were conducted. Ultimately, the segments were examined using a light microscope (BX50-32H01, Olympus).

### Immunohistochemistry (IHC)

The gastric cancer tissue sections that were fixed with formalin and embedded in paraffin were deparaffinized using xylene and hydrated through a series of ethanol (14). Subsequently, the sections were subjected to a 20-min boiling process in an EDTA buffer and allowed to cool to facilitate epitope retrieval. To neutralize endogenous peroxidase, a 3% hydrogen peroxide solution was applied for 15 min. After that, the sections were exposed to primary antibodies at a temperature of 37°C for 1 h. Afterward, the sections were washed and subsequently exposed to suitable biotinylated secondary antibodies at a temperature of 37°C for a duration of 25 min. Finally, the sections were exposed to a diaminobenzidine solution for 2 min and counterstained with hematoxylin. We performed IHC to detect CD45 (MAB-0024, Maxim Biotechnologies, Fuzhou, China), CD19 (MAB-0705, Maxim Biotechnologies, Fuzhou, China), CD4 (ZA-0519, ZSGB Biotechnologies, Beijing, China), CD8 (MAB-0021, Maxim Biotechnologies, Fuzhou, China), CD11c (60258-1-lg, Proteintech), CD68 (ZA-0060, ZSGB Biotechnologies, Beijing, China), and P53 (ZM-0408, ZSGB Biotechnologies, Beijing, China) expression in patient samples.

### Assessment of tertiary lymphoid structures (TLSs) and immunohistochemistry (IHC)

The TLSs underwent morphological assessment on H&E stained slides, which were converted into whole slide images using the Pathological Digital Section Scanning and Application System (Motic, Xiamen, China), as explained earlier (15, 16). Two pathologists, who were unaware of the patient’s clinical information, independently assessed the existence, amount, and variations of TLSs on H&E stained slides. TLS abundance was determined by the patient’s highest number of TLSs observed. Based on the maturation process, TLSs were classified into three categories: (1) Aggregates (Agg), which are small, almost circular groups of lymphocytes; (2) lymphoid follicles I (FL-I), which are large clusters without the formation of germinal centers; and (3) lymphoid follicles II (FL-II), which are lymphoid follicles that have formed germinal centers. In this study, we scored by combining the number or maturity of TLS, with the specific formula being: TLS score = 1*n(Agg) + 2*n(FL-I) + 3*n(FL-II), where n represents the quantity (17, 18).

### Statistical analyses

The ‘survival’ R package was employed to produce the Kaplan–Meier survival graphs and evaluate the OS and DFS. Using the R package “survmine,” patients were subgrouped into low and high TLS scores. To evaluate statistical differences among groups, a two-tailed *t*-test was utilized for normally distributed variables, whereas a one-way ANOVA test was used. To evaluate statistical differences among groups for variables that are not normally distributed, a Wilcoxon test was employed, while the Kruskal-Wallis rank sum test was utilized to determine statistical differences between groups. The statistical analyses were performed in the SPSS (v.22.0), and most of the plots were created with the R software (v.3.6.0).

## Results

### Tertiary lymphoid structures and clinicopathological characteristics of patients

In this retrospective study, paraffin-embedded specimens were collected from 223 surgically treated GC patients. The mean age was 66 years (Range: 21–89 years), and 50 patients (22.4%) were female. According to the histological examination of resected tumors, the differentiation degree of patients was well (75.9%), middle (19.0%), and poor (5.1%). P53 expression was positive in 140 patients (65.4%). The clinical characteristics are listed in Table 1. A total of 212 patients (95.1%) had TLS, and 101 (45.2%) had mature TLS.

**Table 1:** Clinicopathological characteristics of the patients according to the low and high TLS scores.

Characteristics		High TLS scores *n* = 119(53.4%)	Low TLS scores *n* = 104(46.6%)	*p-*value
Age				
	<=70	86(59.7)	58(40.3)	0.012*
	>70	33(41.8)	46(58.2)	
Gender				
	Female	23(46.0)	27(54.0)	0.262
	Male	96(55.5)	77(44.5)	
Differentiation				
	Poor	93(56.7)	71(43.3)	0.136
	Middle	21(51.2)	20(48.8)	
	Well	3(27.3)	8(72.7)	
P53 expression				
	Mutant	60(53.6)	52(46.4)	0.803
	Wild	55(51.9)	51(48.1)	
N				
	N0	59(60.8)	38(39.2)	0.269
	N1	20(46.5)	23(53.5)	
	N2	14(50.0)	14(50.0)	
	N3	26(47.3)	29(52.7)	
M				
	M0	118(53.9)	101(46.1)	0.262
	M1	1(25.0)	3(75.0)	
T				
	T1	41(61.2)	26(38.8)	0.350
	T2	14(50.0)	14(50.0)	
	T3	17(43.6)	22(56.4)	
	T4	47(52.8)	42(47.2)	
Lauren				
	Diffuse	54(61.4)	34(38.6)	0.066
	Intestinal	3(27.3)	8(72.7)	
	mixed	60(51.3)	57(48.7)	
Chemotherapy response				
	No Response	27(46.6)	31(53.4)	0.008*
	Response	61(65.6)	32(34.4)	

*p < 0.05. T, tumor size; N, lymph node metastasis; M, distant metastasis. Some undetected or scattered data had been excluded from the analysis.

**Figure 1:**
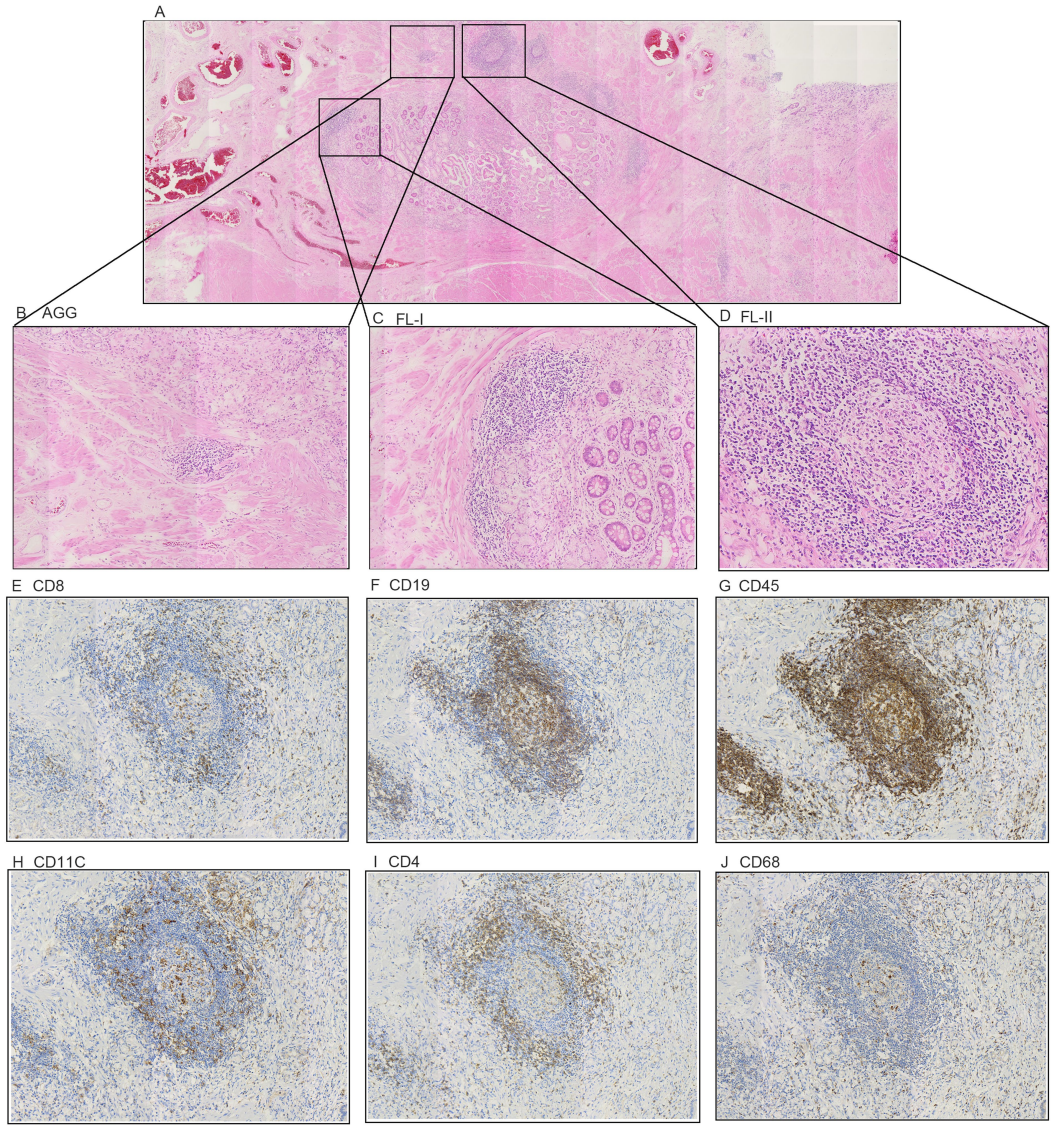
The classification of Tertiary Lymphoid Structures in gastric cancer. **(A)** Representative H&E image of tumor tissue. **(B)** Representative H&E image of TLS Aggregates patients. **(C)** Representative H&E image of TLS Follicles I patients. **(D)** Representative H&E image of TLS Follicles II patients. **(E–J)** Representative IHC image of the cellular composition of TLS. (E) CD8+ T cells. **(F)** CD19+ B cells. **(G)** CD45+ lymphocytes. **(H)** CD11c+ DCs **(I)** CD4+ T cells. **(J)** CD68+ TAMs.

Postnatally, TLSs form in non-lymphoid tissues as organized collections of immune cells, typically seen during chronic inflammation like autoimmune disorders, chronic infection, and cancer (5, 16). When we observed the histopathological characteristics of the tumors in GC samples, we found that TLS was present in a widespread manner (Figure 1). According to the maturity of the TLSs, they were classified as AGG (Figure 1B), FL-I (Figure 1C), FL-II (Figure 1D). To evaluate the presence and maturity of TLS, the general composition of TLSs in GC patients was investigated using conventional immunohistochemistry in serial sections stained for CD8+ T cells (Figure 1E), CD19+ B cells (Figure 1F), CD45+ lymphocytes (Figure 1G), CD11c+ dendritic cells (Figure 1H), CD4+ T cells (Figure 1I), as well as CD68+ macrophages (Figure 1J). Based on previous studies, we scored the combined distribution of TLSs for each sample (6). Patients were assigned to subgroups with low 46.6% (104 of 223) and high 53.4% (119 of 223) TLS scores based on the “survminer” R package. We assessed the relationship between TLS scores and some clinicopathological characteristics such as gender, age, and tumor stage using a chi-square test. As shown in Table 1, TLS scores were significantly associated with age (*p* = 0.012) and chemotherapy response (*p* = 0.008).

### The prognostic values of tertiary lymphoid structures

**Figure 2:**
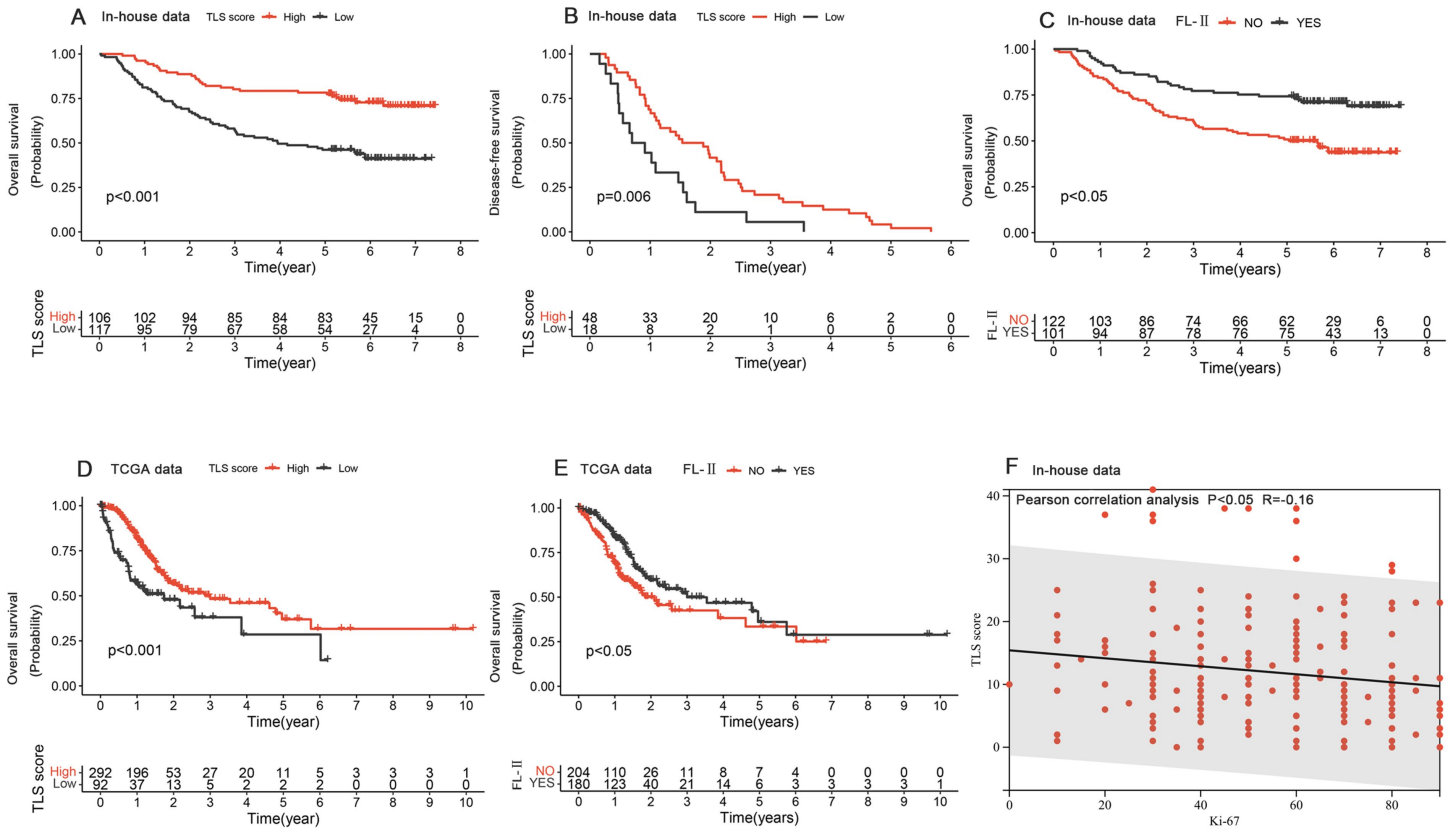
The prognostic values of Tertiary Lymphoid Structures. **(A,B)** Kaplan–Meier curves for overall survival **(A)** and recurrence-free survival **(B)** based on the TLS score in the in-house data. **(C)** The Kaplan–Meier curve of overall survival depended on mature/immature tertiary lymphoid structure in the in-house data. **(D)** The Kaplan–Meier curve for overall survival was based on the TLS score in the TCGA data. **(E)** The Kaplan–Meier curve of overall survival depended on mature/immature tertiary lymphoid structure in the TCGA data. **(F)** The correlation analysis of TLS score and Ki-67 expression.

We evaluated the prognostic value of TLSs in GC patients by constructing Kaplan–Meier curves. The groups with higher TLS scores demonstrated relatively better overall survival and disease-free survival rates among GC patients (Figures 2A,B). Survival analysis revealed that patients with mature TLSs have significantly better survival rates than patients without mature TLSs (Figure 2C). We also validated the prognostic significance of TLS scores and the presence of mature TLS in the TCGA database (Figures 2D,E). We divided the patients into high and low score groups based on the median and then repeated the survival analysis. The results indicated that the high-score group continued to show better prognostic significance (Supplementary Figure S1). Additionally, both univariate and multivariate analyses indicated that TLS score may serve as an independent prognostic factor in GC patients (Table 2). Ki67 was a very valuable indicator of how active tumor cells are, and we found a negative correlation between Ki67 positivity and TLS score (Figure 2F). The results suggested that either high TLS scores or the presence of mature TLSs predicted a better survival rate.

**Table 2:** Univariate and multivariate analysis of prognostic factors for overall survival.

Variable		Univariate analysis		Multivariate analysis	
		*p*-value	OR (95% CI)	*P*-value	OR (95% CI)
Age		<0.001*	1.049(1.026-1.072)	0.126	1.107(0.995–1.038)
Gender		0.949	0.984(0.607–1.597)		
	Female				
	Male				
Differentiation		0.461	1.154(0.789–1.687)		
	Poor				
	Middle				
	Well				
T		<0.001*	2.016(1.644-2.473)	0.002	1.489(1.162–1.907)
	T1				
	T2				
	T3				
	T4				
N		<0.001*	1.648(1.403–1.936)	0.222	1.125(0.931–1.358)
	N0				
	N1				
	N2				
	N3				
M		0.016*	4.151(1.301–13.153)	<0.001*	14.278(4.167–48.916)
	M0				
	M1				
Lauren		0.225	1.249(0.872–1.788)		
	Diffuse				
	Intestinal				
	mixed				
Metastasis		<0.001*	9.558(6.119–14.931)	<0.001*	5.820(3.528–9.602)
	Yes				
	No				
TLS scores		<0.001*	0.939(0.913–0.967)	0.001	0.950(0.921–0.980)

*P < 0.05. T, tumor size; N, lymph node metastasis; M, distant metastasis. Some undetected or scattered data had been excluded from analysis.

### The effects of tertiary lymphoid structures on distant metastasis

**Figure 3:**
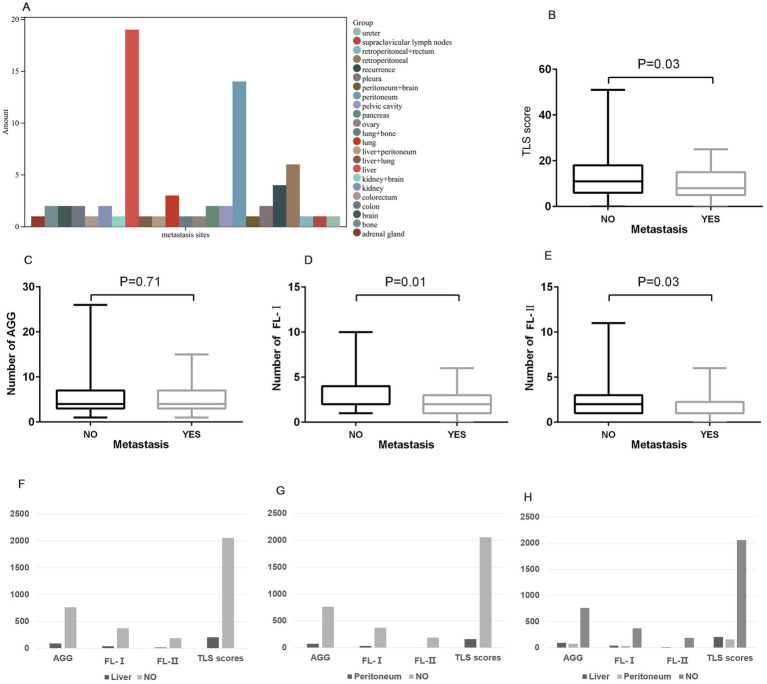
The distribution of Tertiary Lymphoid Structures on distant metastasis. **(A)** The distribution of locations of distant metastases in GC patients. **(B–E)** The distribution of TLS score **(B)**, AGG **(C)**, FL-I **(D)**, and FL-II **(E)** in samples with and without distant metastasis. **(F)** The distribution of various TLSs in samples with liver metastasis compared to samples without liver metastasis. **(G)** The distribution of various TLSs in samples with peritoneal metastasis compared to samples without peritoneal metastasis. **(H)** The distribution of various TLSs in samples among liver, peritoneum, and without metastasis.

In our study cohort, distant metastases were found to occur in 31.8% (71 of 223) of the patients, with a predominance of liver (26.8%) and peritoneal metastasis (19.7%) (Figure 3A). We further analyzed the variation in the distribution and number of TLSs in samples with and without distant metastasis. The results showed a marked difference in the distribution of FL-I (*p* = 0.01), FL-II (*p* = 0.03), and TLS score (*p* = 0.03) in samples with and without distant metastasis, and no statistically significant difference in the distribution of AGG (Figures 3B–E). Compared to samples without distant metastasis, the density of various Tertiary Lymphoid Structures was significantly lower in samples with peritoneal or liver metastasis (Figures 3F–H).

### The prediction role of TLSs in chemotherapy response

**Figure 4:**
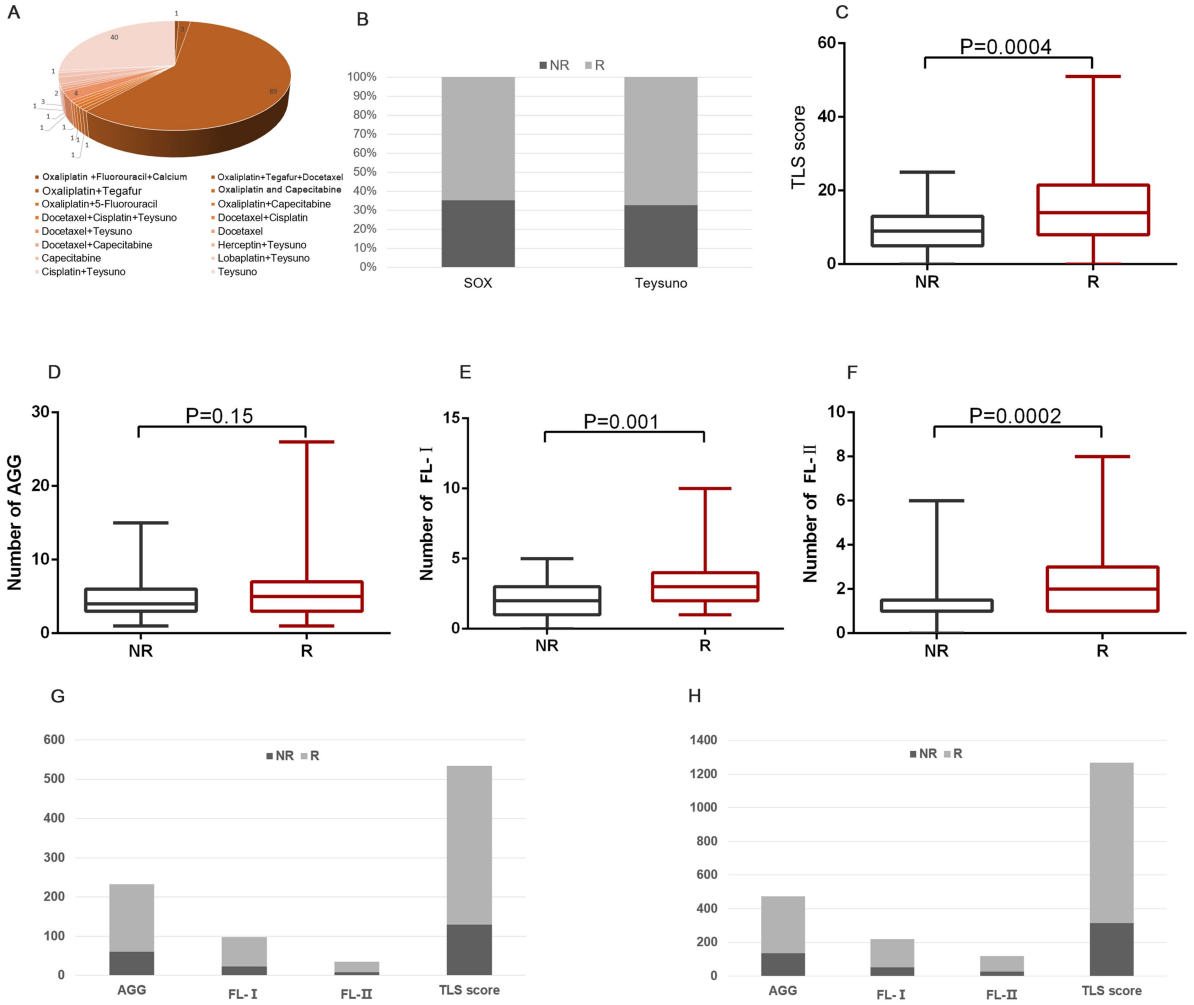
The prediction role of TLSs in chemotherapy response. **(A)** The patient’s medication profile in GC patients. **(B)** The proportion of people responding well to chemotherapy treatment with different drugs. **(C–F)** The distribution of TLS score **(C)**, AGG **(D)**, FL-I **(E)**, and FL-II **(F)** in patients who responded and did not respond to chemotherapy. **(G)** The distribution of various TLSs in patients who responded and did not respond to Teysuno monotherapy. **(H)** The distribution of various TLSs in patients who responded and did not respond to Oxaliplatin and Teysuno therapy.

In our cohort, 68.1% (151 of 223) of these patients underwent adjuvant chemotherapy. The patient’s medication profile was shown in Figure 4A, which was mainly based on the Oxaliplatin and Teysuno therapy or Teysuno monotherapy. Patients responded differently to treatment with different drugs, with 65.1% (58 of 89) responding well to the Oxaliplatin and Teysuno therapy, and 67.5% (27 of 40) responding well to Teysuno monotherapy (Figure 4B). In samples of patients who responded and did not respond to chemotherapy, we analyzed variations in the distribution and number of TLS. The results showed a significant difference in the distribution of FL-I (*p* = 0.001), FL-II (*p* = 0.0001), and TLS scores (*p* = 0.0004) between the samples of patients who responded to chemotherapy and those who did not, and no statistically significant difference in the distribution of AGGs (Figures 4C–F). We further counted the distribution of TLSs in the samples of patients who responded to chemotherapy and those who did not. The density of TLSs was much higher in samples from patients who responded to the Oxaliplatin and Teysuno therapy than in those who did not. Similarly, the density of TLSs was much greater in samples from patients who responded to Teysuno monotherapy than in those who did not (Figures 4G,H).

## Discussion

The understanding of the molecular pathogenesis of tumor has greatly improved due to tremendous sequencing studies (19–22). Nevertheless, given the diversity and adaptability of tumors, it is imperative to categorize individuals with cancer and implement a tailored medication strategy (3). It is crucial to design logically sequential treatment strategies based on each patient’s natural history, tumor histopathology, and molecular tumor characteristics, as well as their chemotherapy response (23).

In studies within lung, colorectal, and pancreatic cancers, it had been shown that the presence of Tertiary Lymphoid Structures (TLSs) density and its components (T follicular helper cells, follicular B-cells, DCs, and high endothelial venules, among others) correlated with better survival in a wide variety of tumor types (10, 24, 25). Moreover, several signature sets associated with TLS had shown positive prognostic predictive value, including plasma cell signatures in ovarian cancer; T follicular helper cell signatures in squamous cell carcinoma of the head and neck; and a variety of gene signatures (including CCL5, CXCL9, CXCL10, and CXCL13) associated with lymph chemokines in colorectal, melanoma, and breast cancer (5, 26, 27). In this study, we retrospectively collected clinicopathological information on GC samples and performed a specialized pathological evaluation to comprehensively assess the distribution of TLSs in each sample. We observed that TLSs were widely present in GC tissues, and patients in the high TLS group experienced improved overall survival and disease-free survival in comparison to those in the low TLS group. TLS was relevant to a long-term prognosis in several cancer types and the prognostic value of TLS was usually independent of TNM staging (9, 10, 28). We analyzed the relationship between TLS and other clinicopathological features, which similarly validated this statement.

Adjuvant chemotherapy has been reported to cause impaired TLS maturation (5). In addition, similar results had been seen after steroid therapy in lung cancer and hepatoblastoma (29, 30). It was not entirely clear whether the negative effects on TLS tissue depended on the type of chemotherapy used. In our analysis, the distributional status of TLS was strongly correlated with the response to adjuvant chemotherapy, and patients with increased numbers and maturation of TLS tended to be more sensitive to chemotherapy. Although adjuvant chemotherapy negatively affects TLS maturation, it enhances patients’ anti-tumor immunity and facilitates treatment response. Previous studies have found that TLS improved immunotherapy outcomes and survival in melanoma and sarcomas (31, 32). Further understanding of the heterogeneity of TLS cellular composition, structural organization, and anatomical location will provide new options for regulating precision tumor therapy (33, 34). In the T-cell zone of TLS, mature dendritic cells, B cells, and effector T cells interact to activate their anti-tumor responses. Within the B-cell zone of the TLS, follicular dendritic cells (FDCs) and B cells interact and subsequently activate and differentiate into plasma cells that produce antibodies (33). TLS formation can be selectively induced by tumor-targeted delivery of chemokines and cytokines involved in TLS formation and B-cell maturation. LTα, LIGHT, and CXCL13 are candidate molecules (33).

There are several limitations in this article. This is a retrospective study based on a clinical sample. We need further prospective validation using cohorts with varied clinicopathologic characteristics and treatments. The study’s sample size may be relatively small, which could limit the reliability of the results. Larger-scale and multi-center studies would be better for validating and generalizing these findings. Direct evidence of the unique nature of the immune responses that develop or are enhanced in TLS is lacking. TLS has been shown to have unquestionable prognostic predictive value in a wide range of cancer types. We lack consistent markers to define and characterize TLS, and this will be a future direction that the field will need to focus on to maximize the value of TLS as a potential biomarker.

## Conclusion

We provided a more comprehensive definition of the “TLS state” spectrum based on histopathologic aspects such as cell composition, number, and maturity. Characterizing the molecular level of TLS status will help to increase the value of TLS as a prognostic predictive marker and will help to fully exploit the potential value of TLS on tumor response to therapy.

## Data Availability

The original contributions presented in the study are included in the article/Supplementary material, further inquiries can be directed to the corresponding authors.

## Footnotes

^1^https://portal.gdc.cancer.Gov/

## References

[1] Li K Zhang A Li X Zhang H Zhao L. Advances in clinical immunotherapy for gastric cancer. Biochim Biophys Acta Rev Cancer. (2021) 1876:188615. doi: 10.1016/j.bbcan.2021.188615 34403771

[2] Zhang M Hu S Min M Ni Y Lu Z Sun X. Dissecting transcriptional heterogeneity in primary gastric adenocarcinoma by single cell RNA sequencing. Gut. (2021) 70:464–475. doi: 10.1136/gutjnl-2019-320368 32532891 PMC7873416

[3] Saleh R Elkord E. Acquired resistance to cancer immunotherapy: role of tumor-mediated immunosuppression. Semin Cancer Biol. (2020) 65:13–27. doi: 10.1016/j.semcancer.2019.07.017, PMID: 31362073

[4] Pernot S Terme M Radosevic-Robin N Castan F Badoual C Marcheteau E. Infiltrating and peripheral immune cell analysis in advanced gastric cancer according to the Lauren classification and its prognostic significance. Gastric Cancer. (2020) 23:73–81. doi: 10.1007/s10120-019-00983-3 31267360

[5] Schumacher TN Thommen DS. Tertiary lymphoid structures in cancer. Science. (2022) 375:abf9419. doi: 10.1126/science.abf9419 34990248

[6] Wakasu S Tagawa T Haratake N Kinoshita F Oku Y Ono Y. Preventive effect of tertiary lymphoid structures on lymph node metastasis of lung adenocarcinoma. Cancer Immunol Immunother. (2023) 72:1823–34. doi: 10.1007/s00262-022-03353-8, PMID: 36688996 PMC10992259

[7] Vanhersecke L Brunet M Guégan JP Rey C Bougouin A Cousin S. Mature tertiary lymphoid structures predict immune checkpoint inhibitor efficacy in solid tumors independently of PD-L1 expression. Nature Cancer. (2021) 2:794–802. doi: 10.1038/s43018-021-00232-6, PMID: 35118423 PMC8809887

[8] Fridman WH Sibéril S Pupier G Soussan S Sautès-Fridman C. Activation of B cells in tertiary lymphoid structures in cancer: anti-tumor or anti-self? Semin Immunol. (2023) 65:101703. doi: 10.1016/j.smim.2022.101703 36481358

[9] Aloisi F Pujol-Borrell R. Lymphoid neogenesis in chronic inflammatory diseases. Nat Rev Immunol. (2006) 6:205–17. doi: 10.1038/nri1786 16498451

[10] Munoz-Erazo L Rhodes JL Marion VC Kemp RA. Tertiary lymphoid structures in cancer – considerations for patient prognosis. Cell Mol Immunol. (2020) 17:570–5. doi: 10.1038/s41423-020-0457-0, PMID: 32415259 PMC7264315

[11] Gutiérrez-Melo N Baumjohann D. T follicular helper cells in cancer. Trends Cancer. (2023) 9:309–25. doi: 10.1016/j.trecan.2022.12.007 36642575

[12] Li Z Jiang Y Li B Han Z Shen J Xia Y. Development and validation of a machine learning model for detection and classification of tertiary lymphoid structures in gastrointestinal cancers. JAMA Netw Open. (2023) 6:e2252553. doi: 10.1001/jamanetworkopen.2022.52553, PMID: 36692877 PMC10408275

[13] Jiang S Ding X Wu Q Cheng T Xu M Huang J. Identifying immune cells-related phenotype to predict immunotherapy and clinical outcome in gastric cancer. Front Immunol. (2022) 13:13. doi: 10.3389/fimmu.2022.980986 PMC9402937 36032097

[14] Jiang S Zhang Y Zhang X Lu B Sun P Wu Q. GARP correlates with tumor-infiltrating T-cells and predicts the outcome of gastric Cancer. Front Immunol. (2021) 12:660397. doi: 10.3389/fimmu.2021.660397, PMID: 34421887 PMC8378229

[15] Horeweg N Workel HH Loiero D Church DN Vermij L Léon-Castillo A. Tertiary lymphoid structures critical for prognosis in endometrial cancer patients. Nat Commun. (2022) 13:1373. doi: 10.1038/s41467-022-29040-x, PMID: 35296668 PMC8927106

[16] Li R Huang X Yang W Wang J Liang Y Zhang T. Tertiary lymphoid structures favor outcome in resected esophageal squamous cell carcinoma. J Pathol Clin Res. (2022) 8:422–35. doi: 10.1002/cjp2.281, PMID: 35711130 PMC9353661

[17] Mao Y Wang X Xi L Dong M Song P Miao J. Prediction values of tertiary lymphoid structures in the prognosis of patients with left- and right-sided colon cancer: a multicenter propensity score-matched study. Int J Surg. (2023) 109:2344–58. doi: 10.1097/JS9.0000000000000483, PMID: 37247038 PMC10442147

[18] Calderaro J Petitprez F Becht E Laurent A Hirsch TZ Rousseau B. Intra-tumoral tertiary lymphoid structures are associated with a low risk of early recurrence of hepatocellular carcinoma. J Hepatol. (2019) 70:58–65. doi: 10.1016/j.jhep.2018.09.003, PMID: 30213589

[19] Dong L Lu D Chen R Lin Y Zhu H Zhang Z. Proteogenomic characterization identifies clinically relevant subgroups of intrahepatic cholangiocarcinoma. Cancer Cell. (2022) 40:70–87.e15. doi: 10.1016/j.ccell.2021.12.006, PMID: 34971568

[20] Lin J Dai Y Sang C Song G Xiang B Zhang M. Multimodule characterization of immune subgroups in intrahepatic cholangiocarcinoma reveals distinct therapeutic vulnerabilities. J Immunother Cancer. (2022) 10:e004892. doi: 10.1136/jitc-2022-004892, PMID: 35863823 PMC9310257

[21] Andersen JB Spee B Blechacz BR Avital I Komuta M Barbour A. Genomic and genetic characterization of cholangiocarcinoma identifies therapeutic targets for tyrosine kinase inhibitors. Gastroenterology. (2012) 142:1021–1031.e15. doi: 10.1053/j.gastro.2011.12.005, PMID: 22178589 PMC3413201

[22] Jusakul A Cutcutache I Yong CH Lim JQ Huang MN Padmanabhan N. Whole-genome and Epigenomic landscapes of etiologically distinct subtypes of Cholangiocarcinoma. Cancer Discov. (2017) 7:1116–35. doi: 10.1158/2159-8290.CD-17-0368, PMID: 28667006 PMC5628134

[23] Zhang Y Zhang Z. The history and advances in cancer immunotherapy: understanding the characteristics of tumor-infiltrating immune cells and their therapeutic implications. Cell Mol Immunol. (2020) 17:807–21. doi: 10.1038/s41423-020-0488-6, PMID: 32612154 PMC7395159

[24] Pimenta EM Barnes BJ. Role of tertiary lymphoid structures (TLS) in anti-tumor immunity: potential tumor-induced cytokines/chemokines that regulate TLS formation in epithelial-derived cancers. Cancers. (2014) 6:969–97. doi: 10.3390/cancers6020969 24762633 PMC4074812

[25] de Chaisemartin L Goc J Damotte D Validire P Magdeleinat P Alifano M. Characterization of chemokines and adhesion molecules associated with T cell presence in tertiary lymphoid structures in human lung cancer. Cancer Res. (2011) 71:6391–9. doi: 10.1158/0008-5472.CAN-11-0952, PMID: 21900403

[26] Messina JL Fenstermacher DA Eschrich S Qu X Berglund AE Lloyd MC. 12-chemokine gene signature identifies lymph node-like structures in melanoma: potential for patient selection for immunotherapy? Sci Rep. (2012) 2:765. doi: 10.1038/srep00765, PMID: 23097687 PMC3479449

[27] Coppola D Nebozhyn M Khalil F Dai H Yeatman T Loboda A. Unique ectopic lymph node-like structures present in human primary colorectal carcinoma are identified by immune gene array profiling. Am J Pathol. (2011) 179:37–45. doi: 10.1016/j.ajpath.2011.03.007, PMID: 21703392 PMC3123872

[28] Goc J Fridman WH Hammond SA Sautès-Fridman C Dieu-Nosjean MC. Tertiary lymphoid structures in human lung cancers, a new driver of antitumor immune responses. Onco Targets Ther. (2014) 3:e28976. doi: 10.4161/onci.28976, PMID: 25083325 PMC4106161

[29] Remark R Lupo A Alifano M Biton J Ouakrim H Stefani A. Immune contexture and histological response after neoadjuvant chemotherapy predict clinical outcome of lung cancer patients. Onco Targets Ther. (2016) 5:e1255394. doi: 10.1080/2162402X.2016.1255394, PMID: 28123901 PMC5213838

[30] Morcrette G Hirsch TZ Badour E Pilet J Caruso S Calderaro J. APC germline hepatoblastomas demonstrate cisplatin-induced intratumor tertiary lymphoid structures. Onco Targets Ther. (2019) 8:e1583547. doi: 10.1080/2162402X.2019.1583547 PMC6492969 31069152

[31] Petitprez F de Reyniès A Keung EZ Chen TW Sun CM Calderaro J. B cells are associated with survival and immunotherapy response in sarcoma. Nature. (2020) 577:556–60. doi: 10.1038/s41586-019-1906-8, PMID: 31942077

[32] Cabrita R Lauss M Sanna A Donia M Skaarup Larsen M Mitra S. Author correction: tertiary lymphoid structures improve immunotherapy and survival in melanoma. Nature. (2020) 580:E1. doi: 10.1038/s41586-020-2155-6, PMID: 32238929

[33] Fridman WH Meylan M Petitprez F Sun CM Italiano A Sautès-Fridman C. B cells and tertiary lymphoid structures as determinants of tumour immune contexture and clinical outcome. Nat Rev Clin Oncol. (2022) 19:441–57. doi: 10.1038/s41571-022-00619-z, PMID: 35365796

[34] Rodriguez AB Engelhard VH. Insights into tumor-associated tertiary lymphoid structures: novel targets for antitumor immunity and Cancer immunotherapy. Cancer Immunol Res. (2020) 8:1338–45. doi: 10.1158/2326-6066.CIR-20-0432, PMID: 33139300 PMC7643396

