# Supplementary material for: Maturity and density of tertiary lymphoid structures associate with tumor metastasis and chemotherapy response
Source: Front Med (Lausanne). 2024 Oct 18;11:1435620. doi: 10.3389/fmed.2024.1435620 (PMC11527684; doi:10.3389/fmed.2024.1435620)
Supplement: Supplementary file 2 [file Image_1.pdf]

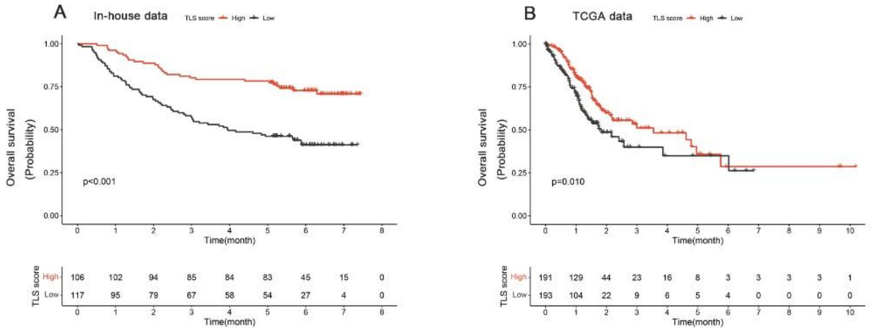

Supplementary Figure 1 The prognostic values of Tertiary Lymphoid Structures. (A) Kaplan-Meier curves for overall survival based on the TLS score in the in-house data. (B) The Kaplan-Meier curve for overall survival was based on the TLS score in the TCGA data.
